# Production of sorbet with persimmon using green pea aquafaba: physicochemical characterization and bioaccessibility of bioactive compounds

**DOI:** 10.1007/s13197-025-06216-z

**Published:** 2025-01-27

**Authors:** Mahmut Kilicli, Kübra Feyza Erol, Esra Akdeniz, Ömer Said Toker, Fatih Törnük, Mustafa Bayram

**Affiliations:** 1 Department of Food Processing, Gaziantep University, Naci Topcuoglu Vocational School, Gaziantep, Türkiye; 2 Hamidiye Faculty of Health Sciences, Department of Nutrition and Dietetics, University of Health Sciences, Istanbul, Türkiye; 3 Chemical and Metallurgical Engineering Faculty, Food Engineering Department, Yildiz Technical University, Istanbul, Turkiye Türkiye; 4 Faculty of Health Science, Department of Nutrition and Dietetics, Cumhuriyet University, Sivas, Türkiye; 5 Faculty of Engineering, Department of Food Engineering, Gaziantep University, Gaziantep, Türkiye

**Keywords:** Sorbet, Green pea aquafaba, Persimmon, Bioaccessibility

## Abstract

The purpose of this study was to valorize green pea cooking water (aquafaba) as a foaming agent in sorbet samples using with persimmon. Significant amount (22.34% ±1.22) of protein was determined in GPA (green pea aquafaba) at 10 Brix as a dry basis. GPA was added to the homogeneous puree mixture in varying concentrations (2.5, – 5, – 7.5, – 10 and 15%) and sorbet was produced in ice cream machine. The results showed that protein and dietary fiber content increased with the increasing of GPA. While the L value increased with increasing of GPA, the overrun improved from 8 to 51%. According to the experimental data, the Herschel–Bulkley model was fitted to samples, and the flow behavior index values varied between 0.29 and 0.37 (*R*^2^ > 0.97). Total phenolic content (TPC) and antioxidant activity (CUPRAC and DPPH) of the resulting sorbet samples were also determined. Moreover, bioaccessibility of phenolics and antioxidant activities were also determined using in vitro digestion. All of the bioactive parameters are positively affected at sorbet samples. Using GPA as a foaming agent improved the overrun as well as bioactive characteristics of the sorbets. It can be said that 10% GPA gives better results, especially when considering overrun and sensory evaluation.

**Supplementary Information:**

The online version contains supplementary material available at 10.1007/s13197-025-06216-z.

## Introduction

Frozen desserts such as ice cream, gelato and sorbet there are some differences between them. They are generally consumed in the summer months and also these products are loved and consumed from 7 to 70 people around the world. Ice cream is a dairy product that is popularly consumed by all age groups, especially in summer due to its nutritional value and cooling effect. It is generally consists of milk, cream, sweeteners, hydrocolloids, emulsifiers and coloring agents (Van Kleef et al. 2002). Gelato, on the other hand, has a similar content to ice cream, but is more specific to Italy. Compared to ice cream, it remains softer as it has a lower fat content, no increase in volume (< 30%) and no hardening process after the freezing device. Therefore, it is served at a lower temperature (-11 °C) than ice cream (-18 °C) when served. It is also flavored with raw ingredients (like fruit) rather than flavors (Marshall et al. 2003).

The other frozen dessert is a sorbet, which is containing fruit puree/juice and sweetening syrup. Sorbet can be accepted as a healthier product due to it is not include an oil-based ingredient such as milk, cream, etc. and only contains fruit puree and aquafaba or egg white (Michalczyk and Kuczewski 2012). It can also be used honey for sweetening. While the most popular of the frozen desserts is ice cream with many studies, but sorbet has been limited in terms of consumption and research. Topolska et al. (2017) used different sources as a fructan (inulin, which is contribute as a prebiotic) in the sorbet they made with strawberries. During the 12-week storage period of strawberry sorbets, there was no significant change in the fructan level in the first 2 weeks. In the following period, fructose, glucose and sucrose levels increased while fructan content decreased. Ledeker, Chambers, Chambers IV, and Adhikari (2012) produced sorbets with 4 different mango species were examined in terms of sensory and textural aspects. After applying pasteurization at 85 °C for 15 s, they added water at a ratio of 1:1 and sucrose to obtain 32 °Brix. Then, they were frozen in the freezing device. Heat treatment had different effects according to the species generally fruity character decreased, increaseed of cooked apperance much of the texture variation was lost. Tommy Atkin’s sorbet was high in green character and lowest caramelized flavor compared to the other cultivars in sorbet.

Malgor et al. (2020) used gluten-free amaranth proteins in lemon sorbet, both functionally (bioactive peptide) and foaming. They also made sorbet using with egg whites as a control. Since the lemon sorbet obtained with amaranth protein contains more protein compared to egg white, the volume increase was also higher. They stated that amaranth proteins can be an alternative to egg white in terms of technological and functionality propertiesand also sensorially aspect. Moreover, lemon sorbet’s bioactive peptides has been showed antithrombotic activity which released after in vitro digestion.

In another study, they made sorbet using bananas and Brazilian juçara fruit (Machado et al. 2021). They also added *Lactobacillus casei* to be a probiotic product. Probiotics has not fallen below 7.9 log cfu/ml during storage. In the bioactive component analyzes, no significant decrease was observed during the 30-day storage period. They also conducted a very comprehensive sensory analysis with 471 children aged 6–10 years. While approximately 70% of the children stated that they liked it very much, the general acceptability increased to 90%.

In the preliminary studies aquafaba has been observed that volume-increasing feature and at the same time, it gains consistency in the sugary environment. Therefore, in this final product prepared with a mixture of aquafaba and persimmon, it was thought that aquafaba could be both a bulking agent and an alternative to thickeners. Both honey and sugar syrup are not used in the product, only the sugar source from the persimmon was satisfied.

Persimmon is rich in bioactive components such as polyphenols, carotenoids and dietary fiber, especially its antioxidant properties (Butt et al. 2015). It contains about 80% water, 16% sugar (mostly fructose and glucose), on the other hand, more sugar than common fruits, 2% dietary fiber (mostly in insoluble form) and the remaining 2% vitamins and minerals. It contains the most magnesium and calcium as a mineral, while vitamin C is at a higher level than the others. However, vitamin C decreases with the progression of maturation, depending on the Krebs cycle (Pérez-Burillo et al. 2018).

This study set out to investigate the usefulness of aquafaba in terms of technological and functionality in the sorbet. The objectives of this research are to determine whether substituting aquafaba for egg white in sorbet production. Moreover, this study is not only substitute of milk dessert for vegans and also produces a frozen dessert with a lower glycemic index since no sugar is used.

## Material-methods

### Materials

Dry green pea (*Pisum sativum*, Russia) and fresh persimmon (*Diospyros kaki*) were obtained from local markets in Istanbul, Turkey. Green pea was selected as aquafaba source based on the preliminary studies conducted on six pulses (chickpea, white bean, kidney bean, green lentil, broad bean and green pea) due to its high foaming properties (foaming capacity).

### Green pea aquafaba (GPA) preparation

Green pea aquafaba (GPA) was obtained as a by-product from canned food production process as described by (Kilicli et al. 2022; Parmar et al. 2016; Stantiall et al. 2018).

### Preparation of persimmon puree and foam

Fully ripened and fresh persimmon (that were sensorially very similar in terms of color and softness were purchased) without any physical defect were selected and finely washed manually under tap water for 5 min. After washing, persimmon’s skin and the seeds were removed manually using a knife. Then, the fleshy parts of the persimmons (after removing the skin and seeds) were homogenized in a large container. The resulting fruit mixture was shredded in a blender for 10 s as 200 g each. Then, green pea aquafaba (GPA) was added to the homogeneous puree mixture in varying concentrations (2.5, – 5, – 7.5, – 10 and 15%) and mixed with a mixer (Tefal Mastermix 425 W) for 4 min (level 5). In addition, all sorbet samples were produced on the same day and from the same sample container. The whipped samples were left to aging for 24 h at + 4 °C. The aged ice cream mixes were whipped at 0 °C for 20 min in the ice cream machine (SAGE- 220–240 V, mixing speed approximately 225 rpm) and were frozen, hardened using a batch freezer (Arcelik, Gebze, Turkey) at − 18 °C for 24 h. The samples were stored in a deep freezer (Arcelik, Gebze, Turkey) at freezing conditions of − 18 °C prior to analysis.

### Determination of physicochemical properties

Protein, pH and dry matter, total sugar and total dietary fiber content of sorbet samples were performed according to modified procedures of AOAC. Color of sorbet samples were determined with a chroma meter (CR-400, Konica Minolta, Japan). The overrun of sorbets were determined by graduated glass beaker. Differential Calorimetry Scanning (DSC) (TA Instrument Q100, New Castle, USA) technique was used to determine the melting behaviour of sorbet samples. Ten–fifteen mg of the samples were put into hermetically sealed aluminum pans and scanned at 5 °C/min over from − 30 to 30 °C.

### Rheological properties (viscosity analyses)

The rheological measurements of sorbet samples were carried out using a rheometer (Anton Paar, MCR-302, Austria) at a temperature of 25 °C with a 50 mm parallel plate at a 0.5 mm gap distance. Analyses were carried out between 0.1 and 100 1/s shear rate values and shear stress. According to the Herschel–Bulkley equation *k*, *n*, and ${\tau _0}$ values were calculated.

### Determination of bioactive compounds of sorbet samples

#### Extraction

Sorbet samples were extracted by methanol water (80:20) for 24 h at room temperature. After extraction, the mixture was centrifuged at 4500 rpm at room temperature for 10 min. The supernatants were filtered using a 0.45 μm filter and analysis were carried out.

#### Determination of total phenolic content

The total phenolic content of the samples was measured according to the method reported by Singleton and Rossi (1965). The results were expressed as mg gallic acid equivalent (GAE)/g.

#### DPPH radical scavenging activity

The *Radical Scavenging Activity* was measured according to the method by Singh et al. (2002). The DPPH radical scavenging activity was expressed as mg TE/g sample.

#### CUPRAC assay

For this purpose, the analysis was conducted by Apak et al. (2007). The antioxidant capacity value for the extracts was calculated using the calibration equation as mg Trolox equivalent (TE)/g sample.

#### In vitro bioaccessibility

In vitro bioaccessibility of the sorbet samples was determined using the 3-stage digestion method and the stock solutions of simulated digestion fluids (SSF: simulated salivary fluid, SGF: simulated gastric fluid, SIF: Simulated intestinal fluid) warmed up at 37 °C described by (Minekus et al. 2014; Kilicli et al. 2022). Determination of total phenolic content, DPPH and CUPRAC values were measured after each in vitro digestion stage. A schematic representation of in vitro gastrointestinal digestion is given in Supplementary Data (S1).

Bioaccessibility index was calculated using the fallowing Eq. (1)

1 $$\eqalign{& Bioaccessibility\>index\>\left( \% \right) \cr & = {{The\>amount\>of\>matter\>in\>IN\>phase} \over {The\>amount\>of\>the\>matter\>in\>IN + OUT\>phase}} \times \>100 \cr} $$

Here’s what each term represents in this formula: IN: The amount of bioactives present in the intestinal phase. OUT: The amount of bioactives that have been absorbed or are available for absorption. IN is the numerator, indicating the fraction of bioactives in the intestinal phase. IN + OUT is the denominator, representing the total amount of bioactives present in both the intestinal phase and the amount absorbed.

The formula calculates the percentage of bioactives in the intestinal phase relative to the total amount of bioactives available (in the intestinal phase plus those absorbed). This helps determine the proportion of bioactives that are accessible for absorption. A higher BI% suggests a greater proportion of bioactives are present in the intestinal phase relative to the total available, which could imply better bioaccessibility. Conversely, a lower BI% may indicate that a significant portion of the bioactives has already been absorbed or is unavailable in the intestinal phase.

### Sensory evaluation

In the sensory evaluation of sorbets; appearance, texture, taste and aroma, melting in the mouth, foreign taste and general acceptability factors were scored in a scale ranging from 1 to 5 points (1: very bad, 2: dislike, 3: not too bad, 4: good and 5: very good).

### Statistical analysis

The study was carried out with two replicates and each analysis was done three times in replicate. ANOVA, the analysis of variance was used to determine if the differences between the parameters of the samples were significant or not (*p*≤0.05).

## Results and discussion

### Physicochemical properties of dry green pea and sorbets

Dry matter content of dried green pea 91,09 ± 0,09 (g/100 g). According to national food composition database (TURKOMP) water content 9,8 (g/100 g) is similar with our reults. The other compounds are protein 19,82, carbohydrate 42,98, total dietary fiber 23,65 and ash 2,6 (g/100 g) TURKOMP (2014). Physicochemical properties of sorbet samples are given in Table 1 and it is seen that the differences between the results are significant (*p* < 0.05). Dry matter content and pH decreased with increasing GPA. The pH of persimmon and aquafaba are 5.90 ± 0.03 and 5.03 ± 0.03, respectively. Protein and dietary fiber content increased with the increasing of GPA. Stantiall et al. (2018) whose study, they found that the protein content in broken yellow peas (aquafaba) was 1.27%, while the rate of insoluble fiber was 1.63%. According to our previous study (Kilicli and Toker 2022), GPA contains approximately 30% protein and 70% carbohydrates on a dry basis. It is seen that the sugar content of the samples is around 12% (Table 1). In 60% juchara + 25% banana-based sorbet, who was found dry matter content was 14%, protein 0.99%, carbohydrates 11.25% and pH 5.17. They also added 5% sugar to the formulation (Machado et al. 2021). Topolska et al. (2017) made a strawberry sorbet at a percentage 50% fruit, while they used 3 different fructan sources and also sugar at different rates. The total sugar content of 3 different sorbets is approximately; It was calculated as 16.71, 20.21% and 20.54%. Comparable results were found by Malgor et al. (2020) who studied lemon juice was approximately 9% in the sorbets obtained using lemon juice, 22% sugar and 68% water were used. Sorbet produced with amaranth protein contained 0.40% protein and 27.4% carbohydrate, while sorbet with egg white had 0.21% and 31% content.

**Table 1:** Physicochemical properties of the sorbet samples

Samples	Dry matter (%)	pH	Protein content (%)	Total sugar (%)	Dietary fiber (%)	L*	a*	b*
2.5	17.53 ± 0.16^ab^	5.54 ± 0.01^a^	0.99 ± 0.02^c^	12.67 ± 0.14^ab^	0.83 ± 0.04^c^	52.84 ± 1.04^a^	8.76 ± 0.67^b^	31.69 ± 1.52^a^
5	17.76 ± 0.20^a^	5.34 ± 0.03^b^	1.06 ± 0.04^bc^	12.86 ± 0.08^a^	1.06 ± 0.03^bc^	55.57 ± 0.88^ab^	8.71 ± 0.43^b^	31.90 ± 2.82^a^
7.5	17.50 ± 0.00^ab^	5.24 ± 0.00^c^	1.14 ± 0.05^abc^	12.08 ± 0.11^c^	1.24 ± 0.06^b^	57.09 ± 2.08^b^	8.79 ± 0.43^b^	35.09 ± 0.72^a^
10	16.92 ± 0.18^bc^	5.10 ± 0.00^d^	1.24 ± 0.06a^ab^	12.29 ± 0.13^bc^	1.66 ± 0.10^a^	60.46 ± 0.54^c^	8.47 ± 0.40^b^	32.84 ± 3.32^a^
15	16.33 ± 0.33^c^	5.03 ± 0.01^e^	1.27 ± 0.03^a^	11.28 ± 0.04^d^	1.73 ± 0.07^a^	65.57 ± 0.96^d^	6.26 ± 0.36^a^	30.53 ± 0.92^a^

Different letters in the column are showed statistically significant by Tukey test (*p* < 0.05). Data are the means ± SD of three replicates

Color is one of the most important quality parameters affecting quality and plays an important role in the sensory evaluation of food products for the consumer. When the color values of the sorbets were examined (Table 1), the L value increased due to high amount of aquafaba (*p* ≤ 0.05). Since the color of persimmon is yellow, the b values were higher than the a values. However, a and b values did not differ within themselves (*p* > 0.05). Although it is accepted in line with consumer requests in other colors other than white in frozen products, the first color that comes to mind is white. In our previous study (Kilicli and Toker 2022), among 6 different pulses (chickpeas, beans, lentils, broad beans, kidney beans and peas) the highest foam brightness belongs to peas, and also the other advantage resist gravitational force which foams remain on the spoon.

**Fig. 1:**
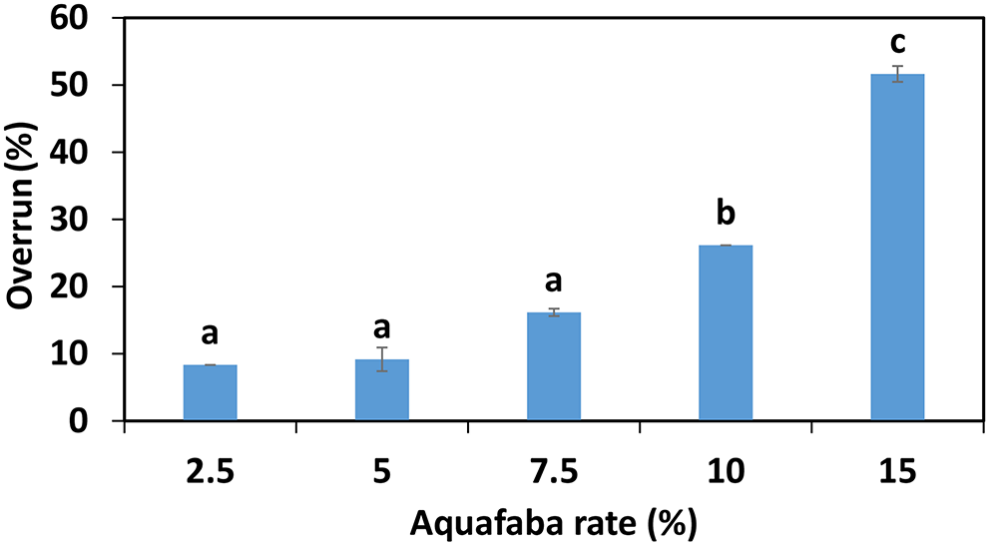
Overrun of sorbets incorporated with aquafaba

The other physicochemical property is the overrun levels of sorbet mixes were presented in Fig. 1. The overrun is desired to frozen desserts, and it is important in terms of the structure of the product and sensory. While the overrun varies between 30 and 50% in a quality sorbet, more overrun can negatively affect as it contains more air in the structure and so may give a feeling of low consistency. The overrun in sorbets were approximately 8.3, 9.2, 16, 26 and 51%, at the rates of aquafaba 2.5, 5, 7.5, 10 and 15%, respectively. As can be seen, the more aquafaba ratio the more overrun. Considering the 30–50% overrun as a quality criterion, it sholud be more appropriate to add 10–15% aquafaba ratio. Malgor et al. (2020) made a lemon sorbet with egg white and a vegetable protein (amaranth). The overrun of the sorbet obtained with egg white was 14%, the other exhibited more as a 36%. While the protein content of the sorbet obtained with amaranth protein was 0.4%, the protein content of the other sorbet (where egg white was used) was found to be 0.21%. In general, egg white has superior properties in foaming because it is an animal protein compared to other plant protein isolates, however it is weak condition may have affected primarily the protein content and also the amino acid composition.

### Flow behaviour of sorbet mixex

**Fig. 2:**
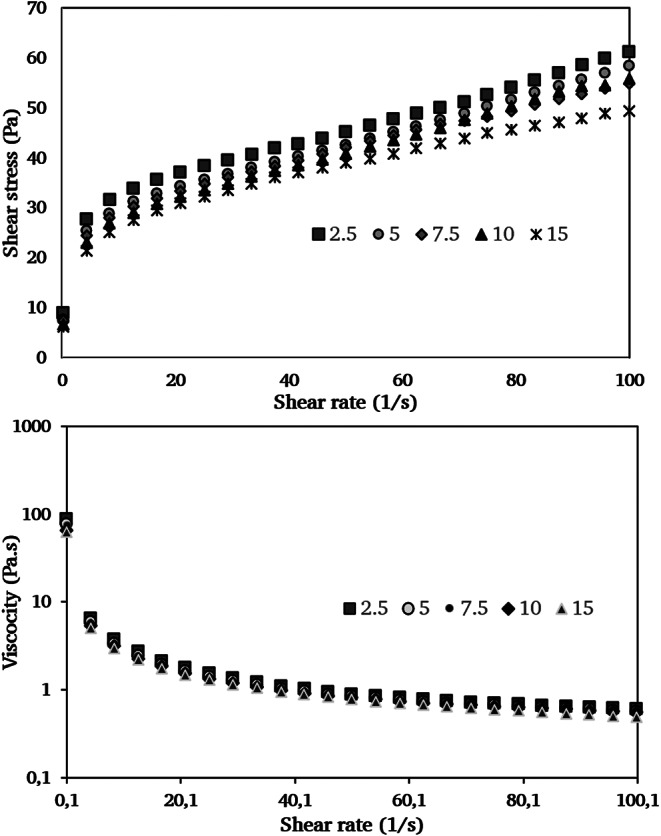
Flow behaviour of sorbet mixes

Flow behavior of sorbet mixes is given in Fig. 2. It is thought that the viscosity of the samples with high persimmon and low aquafaba was higher due to the fibrous structure of the persimmon. While the overrun increased with the higher aquafaba as well as viscosity decreased. There is no significant difference (*p* > 0.05) between the samples in the viscosity curve drawn against the shear rate values. As shown in the figure, shear rate increased higher than the shear stress values, indicating that apparent viscosity decreased with increasing shear rate. This type of flow was known as shear thinning behaviour. According to the experimental data, the Herschel–Bulkley model was fitted to samples, and the flow behavior index values varied between 0.29 and 0.37 (*R*^2^ > 0.97), (Table 2). Similarly, the Herschel-bulkley model was used in the study in which lemon sorbet was produced (Malgor et al. 2020). The yield stress values are given for the samples that do not have a Newtonian. The sample with 15% aquafaba has needed the lowest yield stress, while the sorbet with the highest persimmon and viscosity (2.5% aquafaba) has needed the highest yield stress value. The increase in the air/porous bubbles in the structure as a result of the improving overrun and more aquafaba may also have caused the product to become more fluid. In lemon sorbet study, the viscosity (shear rate = 50) of sorbets produced with amaranth protein was 0.42 Pa.s, while it was 0.11 Pa.s in sorbet using egg white. The viscosity of sorbets produced with persimmon varied between 1.14 and 1.40 Pa.s. The use of lemon juice in the lemon sorbet may have reduced the viscosity. On the other hand the fibrous form of persimmon caused more viscous than lemon juice. The viscosity in shear 50 1/s is called the apparent viscosity of kokini’s and is related to the characterization (thickness) of low-viscosity and mouth-melting foods such as sorbet (Kokini 1987). When the production of sorbets is completed, a viscous structure is obtained, but low viscosity is desired at the beginning for a quick whipping and freezing. Especially sugar syrups added to sorbets produced from fruit juice provide high viscosity, but should be kept at the optimum point during the whipping process.

**Table 2:** Rheological parameters of the Herschel–Bulkley model determined for the sorbet samples

Samples	T_0_ (yield stress)	K (Pa.s^*n*^)	*n*	*R* ^2^
2.5	6.33 ± 0.39^c^	10.85^bc^ ± 0.47	0.34 ± 0.01^abc^	0.974
5	5.86 ± 0.25^c^	9.34^ab^ ± 0.76	0.37 ± 0.02^bc^	0.977
7.5	3.87 ± 0.31^b^	11.26^bc^ ± 0.40	0.32 ± 0.01^ab^	0.987
10	5.91 ± 0.57^c^	7.87^a^ ± 0.65	0.39 ± 0.02^c^	0.982
15	1.33 ± 0.12^a^	12.47^c^ ± 0.99	0.29 ± 0.01^a^	0.995

Different letters in the column are showed statistically significant by Tukey test (*p* < 0.05). Data are the means ± SD of three replicates

### Melting behaviour of sorbet samples

The melting behavior of the samples was determined by DSC and presented in Supplementary Data (S2). Due to the large amount of fruit in the sample containing 2.5% aquafaba (97.5% fruit), the starting to melt (T_onset_) was higher than the others (*p* ≤ 0.05), but no statistical difference (*p* > 0.05) was observed among the others. Considering the T_end_ values, although there are some fluctuations, an increase is seen from the sample 2.5% (aquafaba) to the sample 15% (*p* ≤ 0.05). The melting of ice cream is affected by overrun, emulsifiers, dry matter, structure of ice crystals, oil and protein content (Sofjan and Hartel 2004). In another study the melting rate depend on various factors such as amount of air in the sample, structure of ice crystals, network of oil globules formed during freezing and dry matter content (de Medeiros et al. 2019). In the sorbet samples, there is an increase in the protein content from 2.5 to 15%, as well as an higher overrun. While the lowest ΔH belonged to the 7.5% sample, the maximum energy was recorded in the sample 10%. The amount of air in the sample increased with higher aquafaba, while the dry matter content in the sample decreased with lower persimmon.

Akbari et al. (2016) stated that the higher overrun slows the melting rate of the frozen desserts due to the low thermal conductivity of the air. Therefore higher overrun products require more energy. However, the lower energy value belong to 15% aquafaba compared to 10%. It may be due to more water in the sample in terms of thermal conductivity. Another parameter affecting the melting is the content of pectin in the compound of persimmon, which is approximately 2% (Chen et al. 2016). As the amount of pectin increases, the melting becomes slower (Zhang et al. 2018). Thus, more energy may be needed in sorbets with high persimmon content.

### Sensory evaluation

**Fig. 3:**
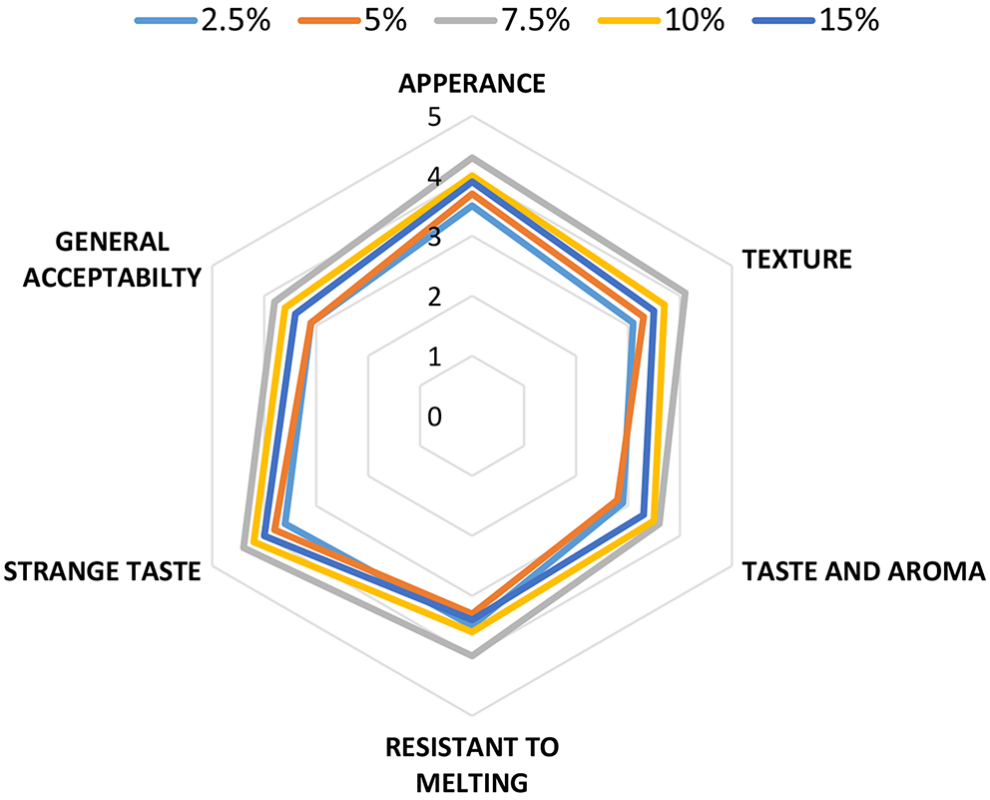
Sensory scores of sorbet samples produced with persimmon and aquafaba

Sensorial characteristics of sorbet products in terms of appearance, texture, taste and aroma, resistant to melting, strange taste and general acceptability are given in Fig. 3. In general, persimmon is a high sweetness fruit, and when aqauafaba was used at 20% in preliminary experiment, it lost its sweetness to a great extent. So aquafaba was used change from 2.5 to 15%. As can be seen in the figure, the maximum scores obtained at 7.5% (aquafaba) sample. Samples containing 2.5 and 5% aquafaba scored lower than the other samples in terms of both sweetness and overall acceptability due to the probably higher sweetness. The sample containing 10% aquafaba received the closest scores to the sample containing 7.5% aquafaba. Sensory analysis of meringues showed low acceptance in terms of taste which made with bean and whole green lentil’s aquafaba. However, high acceptance was determined for chickpea and split yellow pea (Stantiall et al. 2018). In addition, texture is an important parameter at frozen products and meringues. In previous study (Kilicli and Toker 2022) the foam of pea and lentil aquafabas showed higher resistant against to gravity compared to others aquafaba’s foams, which were chickpea, bean, kidney bean and broad bean. The smell that come from some aquafabas origin from cooking water is important in terms of strange taste. Although green lentil foam has a high foaming capacity and a non-fluid foam like pea, the foreign taste is more pronounced in green lentil due to cooking water. Therefore, it would be more appropriate to use green pea aquafaba in products.

In the sorbet study with mango, sorbets and fresh fruits were compared (Kilicli and Toker 2022). In the study, in which sensory analysis was at the forefront, 30 different parameters were used. Cooked and caramelized flavors were found to increase in sorbets compared to fresh fruits. They stated that this may be due to heat treatment. It was observed that the sweetness property was similar between fresh and sorbet, but the overall sweetness was more dominant in sorbets. They also observed that the metallic taste was higher in the sorbets and the hardness/bitterness (originated from phenolic compund) was decreased in the sorbets.

### Bioactive properties and in vitro bioaccesibility of sorbet samples

The total phenolic (TP), DPPH and CUPRAC antioxidant activity results of the samples added to the persimmon purees at a certain rate are given in Table 3. The most of the bioactive components from the samples come from the persimmon and the total content of phenolics in aquafaba is quite low.

**Table 3:**
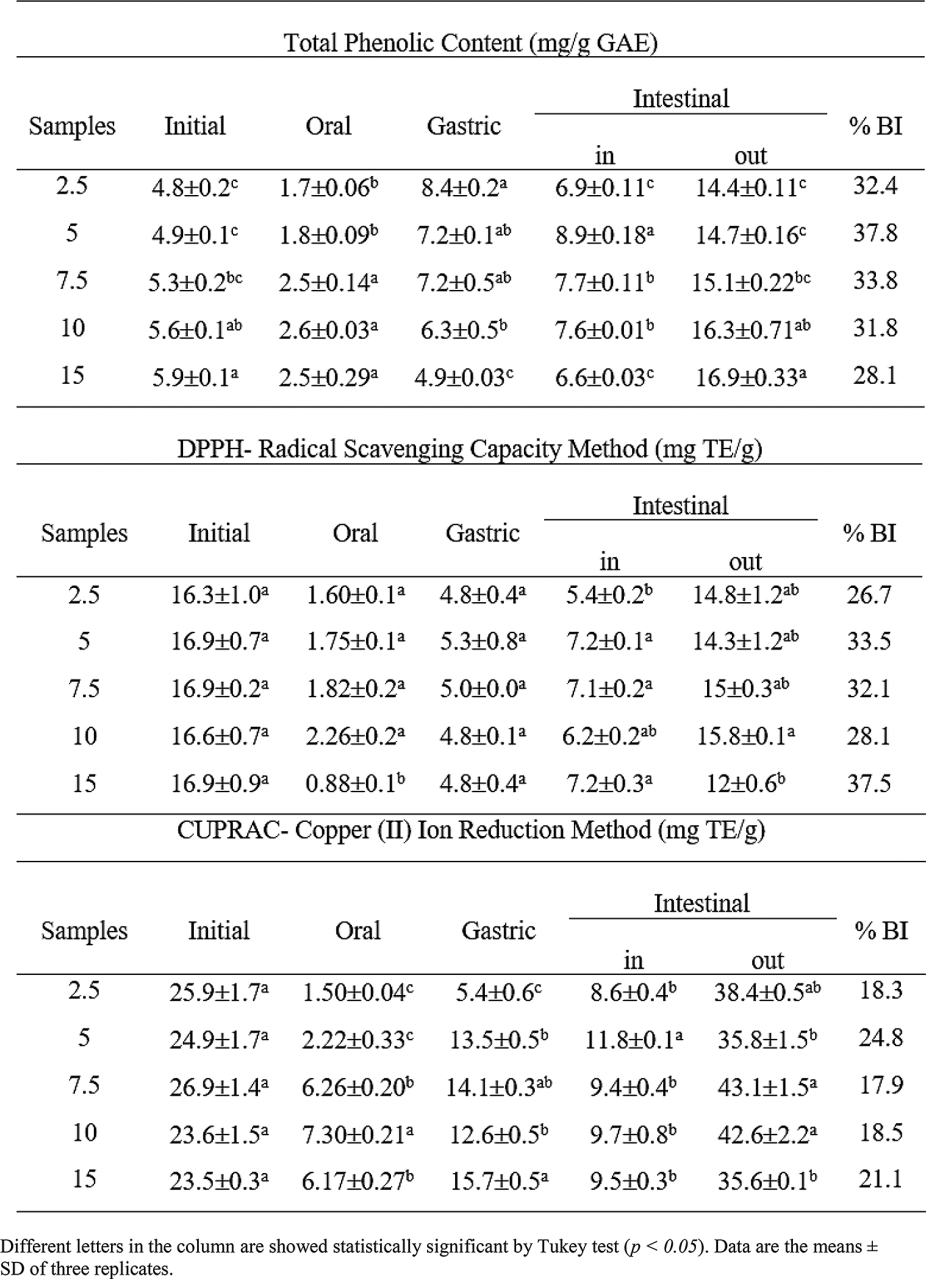
Bioactive compounds of persimmon samples added to GPA at different rates (2.5, 5, 7.5, 10 and 15%) at initial and after digestion

In the study, the TP in the cooking water of pulses was found to be 0.6 mg/g in chickpeas and broken yellow peas. On the other hand, it was found 0.3 in beans and 0.7 mg/g in green lentils (Damian et al. 2018). Persimmon samples were dried with different methods and TP, CUPRAC and DPPH values were found to be 2.65 mg GAE/g, 6.35 and 2.99 mg TE/g, respectively (Kayacan et al. 2020). In this study, the TP was found 5 mg GAE/g, while DPPH and CUPRAC were found 16 and 24 mg TE/g, respectively. Bioactive compounds results may have differed due to fruit type, maturity and extraction method. At the initial stage, TP significantly increased (p *≤ 0.05)* but no significant change was observed in the antioxidant activities (p *> 0.05)* meanwhile persimmon decreased aquafaba increased. The increasing in TP partially from aquafaba and also the contribution of the porous structures can be considered formed by the foaming capacity of aquafaba. Machado et al. (2021) made sorbet with banana and juchara fruit was stored for 30 days for bioactive compound analysis. According to the results, while the total content of phenolic substance did not change, there was increase in DPPH, decrease in FRAP, but did not change in ABTS. This difference in antioxidant results is due to the fact that each method shows a different mechanism of inactivating each free radical (Brand-Williams et al. 1995). Therefore, different antioxidant methods are needed. They attributed the decrease in antioxidant activity (FRAP) to the reducing in Fe^+ 3^ ion, which weakens during storage. There was an improvement at DPPH activity approximately 60%. They stated that the conversion of existing anthocyanins to aglycon forms by enzymes such as beta glucosidase, even at temperatures as low as -80 °C, may cause an increase in antioxidant activity (Hussain et al. 2016).

In terms of bioactive compounds of persimmon samples added to GPA at different rates (2.5, 5, 7.5, 10 and 15%), after intestinal in-phase, all samples had lower level of another digestion phases. The lower values of phenolics in the oral phase, 2 min of digestion with alpha-amylase, in all samples can be explained by the low solubility of these compounds in salivary fluid and the short duration (Ucar and Karadag 2019) of this digestion step similarly as explained in the work, foam-mat drying (Kilicli et al. 2022). The following increase of phenolics, when exposed to stomach conditions, suggests that acidic pH and gastric enzymes may facilitate the release of phenolics bound to the proteins and carbohydrates from different foods. Podio et al. (2019) stated that pepsin in addition to gastric acid condition would promote the break of covalent bonds between polyphenols and the proteins from the food matrix, increasing the phenolic content. There has also been an increase in the transition from stomach to intestinal phase, and we see this situation in other studies (Ucar and Karadag 2019). It can be considered that some compounds are resistant to acidic condition and release at a higher rate in alkaline condition. In addition, the intermediate products formed by Maillard and the alkali medium which providing a better H^+^ donor may have increased of antioxidant activity (Bressa et al. 1996). In Supplementary Data (S3), the bioaccessibility index (BI) values of total phenolic (TF) and antioxidant activity results versus aquafaba are given. While the total phenolic BI value was higher than antioxidant activity BI value up to 15% aquafaba, the antioxidant activity BI values were more than phenolic BI value in 15% aquafaba. The intermediate products’ formed by Maillard reaction of the proteins which including in aquafaba and the sugars from the persimmon may have increased the antioxidant activity. Probiotic bacteria added about 8 logs to produce probiotic sorbet did not show a significant decrease in storage period, while it decreased by about 5 log in the first two hours in in vitro digestion and below 3 log cfu/ml when digestion was finished (Machado et al. 2021). In another study, they investigated the antithrombotic activity of lemon sorbet produced using amaranth protein after in vitro digestion (Malgor et al. 2020). When 3.4 mg/ml amaranth protein is used, an antithrombotic activity is 50%, while when approximately 4 mg/ml is used, an activity of more than 80% has been achieved by the release of bioactive peptides (proteolysis of bioactive peptides found in amaranth proteins).

## Conclusion

In recent years, it has been observed that the demand for herbal food instead of animal food and/or animal-based semi-finished products has increased not only by the vegan population but also throughout the world. This orientation can be for health reasons as well as for economic reasons. Considering in this context, both pulses (in terms of nutrition) and aquafaba (egg replacer) can be an important alternative. It has been seen that aquafaba can be obtained without any nutritional or physical damage to the canned product and its functional properties can be benefited. In addition, GPA, which has superior foaming properties, was successfully used instead of chickpea aquafaba in the study. It has been revealed that aquafaba can be used as an alternative to egg white in sorbet production, which is a product that can be substituted for ice cream in terms of cost and vegan. It can be said that 10% GPA gives better results, especially when considering overrun and sensory evaluation. Better results may be obtained in future studies, perhaps by adding a little sugar.

## Electronic supplementary material

Below is the link to the electronic supplementary material.

Supplementary Material 1

## Data Availability

The data that support the findings of this study are available from the corresponding author, upon reasonable request.
