# Supplementary material for: Production of sorbet with persimmon using green pea aquafaba: physicochemical characterization and bioaccessibility of bioactive compounds
Source: J Food Sci Technol. 2025 Jan 27;63(2):392–400. doi: 10.1007/s13197-025-06216-z (PMC12926268; doi:10.1007/s13197-025-06216-z)
Supplement: Supplementary file 1 — Supplementary Material 1 [file 13197_2025_6216_MOESM1_ESM.docx]

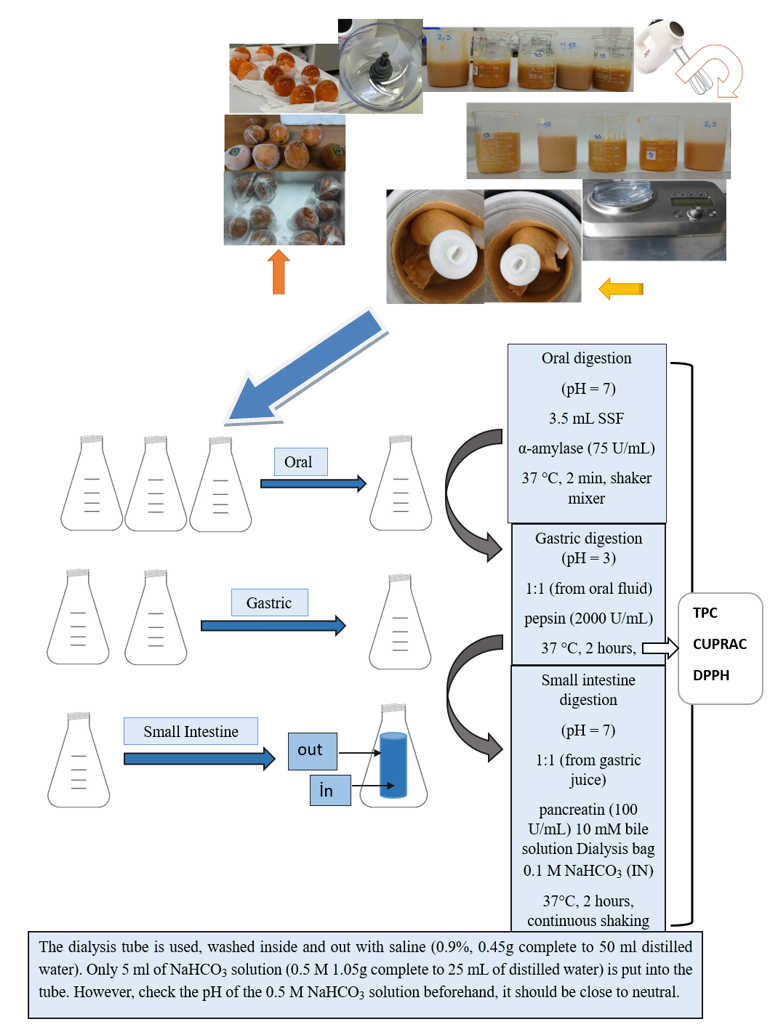
**S1.** A schematic presentation of production of sorbet and flow diagram of simulated *in vitro* gastrointestinal digestion

**S2.** Melting properties of sorbet samples.

| Samples | T_onset_  (°C) | T_end_  (°C) | ΔH  (J/g) |
| --- | --- | --- | --- |
| 2.5 | -12.34 ± 0.07^b^ | 6.74 ± 0.30^a^ | 178.1 ± 0.57^bc^ |
| 5 | -10.69 ± 0.16^a^ | 7.35 ± 0.04^ab^ | 183.8 ± 4.67^cd^ |
| 7.5 | -10.32 ± 0.37^a^ | 6.90 ± 0.08^ab^ | 168.9 ± 8.41^a^ |
| 10 | -10.29 ± 0.04^a^ | 7.56 ± 0.11^b^ | 186.6 ± 6.29^d^ |
| 15 | -10.80 ± 0.08^a^ | 7.51 ± 0.19^b^ | 173.5 ± 0.21^ab^ |

Different letters in the column are showed statistically significant by Tukey test (*p < 0.05*). Data are the means ± SD of three replicates.

**S3.** BI values according to aquafaba ratio at TP, DPPH and CUPRAC**.**
